# Working hours and cardiovascular disease

**DOI:** 10.5271/sjweh.4156

**Published:** 2024-04-01

**Authors:** Reiner Rugulies

**Affiliations:** National Research Centre for the Working Environment (NFA) and Department of Public Health, University of Copenhagen, Copenhagen, Denmark. [email: rer@nfa.dk]

Working hours, including the number and the arrangement thereof – such as shift work, night work, and quick returns – are classic topics in research on work environment and health. The struggle for working time reduction and the eight-hour work day is also one of the oldest fights of the labor movement, dating back to the 19^th^ century (1). International Workers’ Day, celebrated annually on 1 May, has its origin in the *Haymarket Affair*, a rally in support of a strike for the eight-hour work day at the Haymarket Square in Chicago, USA, on 4 May 1886. At the rally, a riot broke out and a bomb exploded, killing several workers and police officers. In the aftermath, the State of Illinois prosecuted labor movement activists. Although the person who throw the bomb was never identified and the circumstances of the attack remained unclear, four labor movement activists, including August Spies, the editor of the German-American newspaper *Arbeiter-Zeitung*, were executed by hanging on 11 November 1887. A fifth activist died by suicide in prison (2).

When the International Labour Organization (ILO) was established as an agency of the newly created League of Nations (the predecessor of today’s United Nations) after World War I in 1919, one of its main aims was the regulation and reduction of working time (1). The demand for the 8-hour work day and 48-hour work week was even included in the peace treaty of Versailles (Part XIII, Section II, Article 427) that was signed on 28 June 1919 following World War I (3, 4).

Although working hours have been greatly reduced in many high-income countries since the 19^th^ century, particularly in Europe, the discussion about working hours remains topical, as can be seen by the recent debate about a 4-day working week (5, 6). In South-East and East Asian countries, such as Japan, South Korea, and Taiwan, where working hours >48 hours per week are still widely prevalent, health concerns of such long working hours are an important topic of discussion (7, 8). Notably, the Japanese language has coined two terms: *karōshi* for death due to overwork (usually of cardiovascular causes) and *karōjisatsu* for death by suicide due to overwork (9). At the *Scandinavian Journal of Work, Environment & Health*, we regularly receive papers from researchers in Asia examining the health effects of long working hours (10–12).

This issue includes a paper from a German research group on the association between night shift work and risk of cardiovascular disease (13). I use this opportunity to reflect briefly in this editorial on research on working hours and cardiovascular health. In the May issue (number 4) of the Journal, as part of our 50-year anniversary special publication series (14, 15), there will be a much more detailed account on what we have learned so far on working hours and health.

## Long working hours and cardiovascular disease

In 2015, Kivimäki et al (16) published a seminal paper on long working hours and cardiovascular disease for the *Individual Participant Data Meta-Analysis of Working Population (IPD-Work) Consortium* that showed an association of long working hours with an increased risk of both ischemic heart disease and stroke (16). The association was stronger for stroke than ischemic heart disease (pooled relative risks 1.33 versus 1.13). Furthermore, for stroke, but not ischemic heart disease, the analyses suggested an exposure–response pattern. Thus, the longer the working hours, the greater the risk of stroke.

From 2017 to 2021, the World Health Organization (WHO) and ILO conducted a project on the WHO/ILO Joint Estimates of the Work-related Burden of Disease and Injury (17, 18) that included systematic reviews on the association of long working hours and risk of ischemic heart disease (19) and stroke (20). Results were similar to those reported by the *IPD-Work Consortium* in 2015. Long working hours, defined as ≥55 hours per week, were associated with a small increased risk of ischemic heart disease (pooled risk ratio 1.17) (19) and a larger increased risk of stroke (pooled risk ratio 1.35) (20). Based on these risk estimates and estimates on the country-specific prevalence of long working hours, the WHO and ILO estimated that, in 2016, globally 745 194 deaths were attributable to long working hours, with the largest burden in South-East Asia (17, 18, 21). A summary of the WHO/ILO joint estimates project has been published as a discussion paper in our Journal (22), together with an editorial (23).

Obviously, the estimate of approximately 750 000 annual deaths due to long working hours is based on several assumptions, including that the epidemiological studies' estimates indicate a causal association between long working hours and cardiovascular outcomes and that data on the worldwide prevalence of long working hours are valid. Kivimäki and colleagues (24) expressed concerns about the interpretation that there is sufficient evidence for harmfulness of long working hours with regard to ischemic heart disease. Among other things, the authors were concerned about residual confounding (eg, by health-related behaviors, although it is debated whether they are mainly confounders, for which one should control, or mediators, for which one should not control (25)). They also presented analyses of data that suggested that socioeconomic position might be an important effect modifier and that the harmful effect of long working hours on risk of ischemic heart disease may be limited to workers of low socioeconomic position. As socioeconomic position is linked to the type of work the workers are doing, the possible effect modification by socioeconomic position could also mean that other, unmeasured working conditions may modify the association between long working hours and health. In other words, in addition to the length of the working hours, what happens during these working hours might also be important.

In Denmark, Hannerz and colleagues (26, 27) attempted to replicate the analyses on long working hours and ischemic heart disease and stroke using large-scale register data. With regard to ischemic heart disease, they did not find an increased risk with long working hours [rate ratio (RR) 1.07, 95% confidence interval (CI) 0.94–1.21 for >48 versus 32–40 hours] (26). When stratified by socioeconomic position, long working hours were not associated with an increased risk of ischemic heart disease among workers of high, medium and unknown socioeconomic position, but there was an increased risk among workers of low socioeconomic position that worked long hours (RR 1.27, 95% CI 1.05–1.53). Although the interaction 'long working hours × socioeconomic position' was not statistically significant, this increased risk among workers of low socioeconomic position is in agreement with the analyses by Kivimäki et al (24). With regard to stroke, Hannerz et al (27) did not find an increased risk for all types of stroke combined among those with long working hours, however, they reported an association between long working hours and increased risk of hemorrhagic stroke. This result was recently replicated in an analysis of the French CONSTANCES study (28) where exposure to long working hours during the past ten years was, in the adjusted model, associated with an increased risk of hemorrhagic stroke but not ischemic stroke.

One can summarize that during the past ten years, several large-scale studies and meta-analyses on long working hours and cardiovascular outcomes have been published. Whereas the WHO has concluded that there is sufficient evidence for harmfulness for the association between long working hours and ischemic heart disease and stroke (19–21), other studies point to possible effect modification by socioeconomic position (24, 26) with regard to ischemic heart disease and the need to distinguish between ischemic and hemorrhagic stroke (27, 28).

## Shift work, night shift work and cardiovascular disease

In 2018, Torquati et al (29) published a systematic review and meta-analysis that showed an increased risk of cardiovascular disease among shift workers. For those working shifts for five years, each additional five-year period of shift work was associated with a 7% increased risk of cardiovascular disease.

Night shift work has been of particular interest for cancer research (30) but might also be relevant with regard to cardiovascular diseases. In their recent review and meta-analysis, Su et al (31) reported that night shift work was associated with an increased risk of cardiovascular mortality (pooled estimate 1.15, 95% CI 1.03–1.29). However, only four studies were included in this review.

In 2022, a Swedish research group published two papers on night shift work from a large sample of healthcare workers in Stockholm, one on cerebrovascular disease and the other on ischemic heart disease. Bigert et al (32) reported that frequent night shifts and frequent consecutive night shifts were associated with an increased risk of cerebrovascular disease, including stroke. Kader et al (33) reported that permanent night shifts and frequent night shifts were associated with an increased risk of ischemic heart disease. In Denmark, Vestergaard et al (34)examined the association between night shift work and ischemic heart disease in a large-cohort of healthcare workers with day-to-day payroll information. The results were less clear than those of Kader et al (33): male, but not female, healthcare workers with night work had an increased risk of ischemic heart disease compared to day-time workers.

The paper by Jankowiak et al (13) in the current issue of the Journal examined night shift work and risk of cardiovascular disease in a population-based cohort in the city of Mainz and Mainz-Bingen in Germany (13). The hazard ratios for low, middle, and high night shift work were 1.19, 1.32, and 1.14, respectively, compared to no night shift work, in the most-adjusted model. An important strength of the study is the comprehensive clinical examination of the participants, both at baseline and follow-up. An important limitation is the very low number of cases in the exposure groups during the five-year follow-up. Unsurprisingly, the CI of all estimates were wide and included unity and, thus, were far away from being statistically significant. The uncertainty of the estimates do not allow firm conclusions on the results. However, the estimates from this study can be included in meta-analyses, which then may provide us with more insight on the role of night shift work on risk of cardiovascular disease.

Important challenges for future research on working time and cardiovascular disease will include better use of electronic working time registration systems that will allow a more precise measurement of exposure to long working hours and the frequency and type of shift work and night shift work (35, 36). It will also be important to conceptually clarify whether health behaviors that are hazardous to cardiovascular health – such as certain dietary patterns, lack of leisure time physical activity, or smoking – are confounders or mediators, or both, for the association between working time and cardiovascular disease. This clarification is key to correctly handling data on these health behaviors in the statistical models. Finally, examining the relations of the different working time arrangements with the contents of work conducted during these arrangements might be fruitful for a better understanding of the contribution of work to cardiovascular disease.
